# Housing-related companion animal relinquishment across 21 animal shelters in the United States from 2019–2023

**DOI:** 10.3389/fvets.2024.1430388

**Published:** 2024-07-10

**Authors:** Jennifer W. Applebaum, Lauren Loney, Kevin Horecka, Taryn M. Graham

**Affiliations:** ^1^Department of Environmental and Global Health, University of Florida, Gainesville, FL, United States; ^2^American Pets Alive!, Austin, TX, United States; ^3^Independent Researcher, Toronto, ON, Canada

**Keywords:** companion animals, housing, relinquishment, animal shelters, pets

## Abstract

Housing issues are a major contributor to companion animal relinquishment in the United States and beyond. In this study, we analyze a database of shelter intake records from 2019–2023 from 21 shelters across the United States to assess rates and subtypes of housing relinquishment, characteristics and outcomes of the relinquished animals, and longitudinal trends in housing relinquishment. Housing issues represented 14% (*n* = 28,424) of overall intakes in the broader database (*N* = 1,021,204 total intake records). Housing relinquishment subtypes were unspecified (54%), pet-related restrictions (27%), landlord issues (8%), housing loss (5%), and unhoused owners (5%). Large (mean weight: 55 lbs) and small dogs (mean weight: 11 lbs) were most common. Pit bull-type dogs comprised 12% of the overall relinquishments and mixed-breed dogs were 35%. Most animals had a live outcome, but live outcomes decreased over time (*p* < 0.001, *z* = −6.91, *slope* = −0.11), and pit bull-type dogs (*X*^2^(1) = 243.63, *p* < 0.001) and animals relinquished by unhoused owners (*OR* = 0.64, *p* < 0.05) were most at risk of euthanasia or other shelter death. Over the study period, intakes due to loss of home increased (*p* < 0.001, *z* = 9.82, *slope* = 0.29), while intakes due to pet restrictions (*p* < 0.001, *z* = −6.82, *slope* = −0.17) and landlord issues decreased (*p* < 0.001, *z* = −4.89, *slope* = −0.08). Overall cat intakes increased (*p* < 0.001, *z* = 3.60, *slope =* 7.34), while dog intakes decreased (*p* < 0.001, *z* = −4.89, *slope* = −0.08). The number of intakes that were pit bull-type dogs (compared to all other breeds) decreased over time (*p* < 0.001, *z* = −4.56, *slope* = −0.06), as did average animal weight (*p* < 0.001, *z* = −4.42, *slope* = −0.07) and age (*p* < 0.001, *z* = −7.88, *slope* = −0.16). We discuss these findings in the context of the previous shelter and pet-friendly housing research and broader housing trends and policies in the United States.

## 1 Introduction

Pets are considered important family members. Their companionship can help benefit human health; however, pet-related restrictions, bans, or surcharges in rental housing can have negative impacts (1–4). Research suggests only approximately 7–9% of all rental housing in the US is free of major pet restrictions (5). The inability to find pet-inclusive housing can result in people sacrificing their health and safety to keep their pets. By contrast, giving up a pet to secure housing can have negative mental health impacts (6). Socially and economically marginalized populations (e.g., low-income households; aging adults; Black, Indigenous, and People of Color (BIPOC)) may especially be impacted by pet restrictions in housing and surcharges (1, 7, 8), as can those caring for large dogs or dogs of certain breeds (2, 3).

In the US, laws and regulations regarding pets in rental housing vary. In market-rate housing, there is no specific legislation prohibiting landlords from banning pets and restricting them based on breed. However, tenants in public housing and housing for the elderly or persons with disabilities subsidized by the Department of Housing and Urban Development [HUD] must be allowed to keep “common household pets” subject to “reasonable” pet policies (9). In California, subsidized rental housing funded through state housing funds requires landlords to allow at least one companion animal per household (10). In subsidized housing under the purview of Public Housing Authorities [PHAs], Texas and Florida recently passed laws prohibiting pet policies from including breed restrictions. In both cases, this legislation requires PHAs to have pet policies in compliance with local dangerous dog laws, which in those states, prohibit identifying a dog as dangerous based on its breed (11, 12). Colorado is the only state with legislation limiting landlords’ ability to require additional nonrefundable fees or rents for pet owners in market-rate housing (13), although at least two municipalities have limited landlords’ ability to charge nonrefundable fees related to pets: Seattle, Washington (14) and Tacoma, Washington (15). Additionally, unrelated to pet ownership, some states limit the use of nonrefundable up-front fees for any reason other than application fees in all rental housing [see, e.g., (16)]. Insurance companies in the US may charge extra based on the dog’s size or breed; however, research shows that damage caused by pets in housing is relatively low (17) and that landlords and property managers can mitigate concerns by implementing pet agreements that outline the responsibilities of pet owners (5). Landlords who establish open lines of communication, reasonable pet-related rules, and who engage in proactive pet-related discussions often experience positive relationships with their tenant (2, 3). Where possible, providing amenities such as designated pet areas can also enhance tenant satisfaction, promote an overall sense of community, and help reduce any problem behaviors (1, 2, 5, 18).

While circumstances leading to companion animal relinquishment are often multifaceted (19), few studies have analyzed animal shelter data to determine the frequency and type of housing-related intakes and related outcomes, and these studies have been limited geographically and/or may be outdated (20–24). Thus, further research is needed to both extend and update previous studies considering the potential impact of more recent trends in housing insecurity and animal sheltering.

### 1.1 Setting

In the United States, approximately 66%, or 86.9 million, households have at least one pet (25–27) and more than 35% of households rent (28). The Humane Society of the United States [HSUS] estimates that 72% of renter households own at least one pet (29). Thus, an estimated 21.9 million households with one or more pets are subject to inconsistent, wide-ranging public and private pet policies in the rental housing market.

Rental costs have surged since 2019, and unaffordability in the rental market hit an all-time high in 2022, according to a 2024 America’s Rental Housing Report by the Joint Center for Housing Studies of Harvard University. HUD defines housing as “affordable” when a household spends no more than 30% of its monthly income on housing-related expenses, including rent and utilities. A household is considered moderately rent-burdened if they spend more than 30% of their monthly income on housing expenses and severely rent-burdened if they spend 50% or more of their monthly income on housing expenses. Harvard’s Joint Center for Housing report found that half of all US renters were moderately cost-burdened, a 3.2% increase from 2019 (30). While this report noted that all income ranges saw increases in rent burden, middle-income renters experienced the highest relative increases in moderately rent-burdened households between 2019 and 2022. Eighty-three percent of low-income renters (those making $30,000 per year or less) were rent-burdened in 2022, with 65% of those households categorized as severely rent-burdened (30). A 21% increase in median rent between 2001 and 2022 vastly outpaced median increases in renters’ incomes, which increased by only 2% in the same time frame (30). Further, a 2024 report by the National Low Income Housing Coalition revealed a shortage of 7.3 million affordable and available homes for extremely low-income renters (31). Both of these reports indicated that moderate and severe rent burden and lack of access to affordable housing are continuing to most impact BIPOC-headed households; a long-understood trend resulting from both historic (i.e., redlining) and current-day racial inequities in access to higher-paying jobs, education, and important credit opportunities, such as mortgages.

Notably, none of these statistics include the ways in which monthly pet rent – a now commonplace pet policy – impacts whether a household is rent-burdened (1). The Humane Society of the United States estimates that more than 20 million pets live with families in poverty (32) and despite a moderate “cooling” of rent prices between 2019 and 2022, more households than ever are struggling to afford housing. These findings underscore the need for policies and solutions to address housing affordability challenges across the US, including for pet owners who rent (1).

### 1.2 The current study

This paper aims to describe the relationship between housing-related intakes and the resulting outcomes of animals in the context of state-level housing policy. What follows in this paper is an analysis of over 28,000 housing-related relinquishment records between 2019–2023 from 21 shelters across the United States. Specifically, we describe:

1. The proportion of intakes related to housing issues.
2. The characteristics of the animals that were relinquished due to housing issues.
3. The outcomes of the housing-relinquished animals.
4. Longitudinal trends in housing intakes by intake subtype, species, size, and (for dogs only) breed.

## 2 Materials and methods

### 2.1 Data

Human-Animal Support Services [HASS] is a national collaborative of municipal shelters and nonprofit rescues whose mission is to keep people and their pets together, reduce euthanasia of healthy, adoptable pets, and facilitate critical engagement of animal welfare organizations, communities, and human services organizations to build a system to better address the modern needs of communities, both for people and the pets they love. HASS has been collecting intake and outcome data for pets entering 21 of these shelters (“pilot shelters”) across the United States since 2019. In 2023, total shelter intake for all pilot shelters ranged from 1,026 to 41,047 animals. Across shelters, the median total intake in 2023 was 8,804 animals. Approximately half of shelters are nonprofit organizations with a government contract providing various elements of municipal animal services. The remainder are municipal/government animal shelters. While most of the shelters serve a single jurisdiction, several serve multiple cities or counties. Pilot shelters were selected based on voluntary interest in working with organizations and individuals in their communities to provide a range of services and solutions to support people and animals. These include remote services like veterinary telehealth and text support, lost animal return-to-owner initiatives, foster care programs, behavioral and training services, and more. Pilot shelters vary greatly in budget, geographic jurisdiction served, human population size served, and intake numbers. Pilot shelters also vary greatly in day-to-day programs and processes. However, all pilot shelters are either municipal animal shelters or nonprofit animal rescues with a municipal contract to provide some or all animal services in that jurisdiction. No additional information about data entry protocols or staff training were available to the researchers. Between November 2019 and December 2023, HASS collected 1,021,204 intake records, representing intakes from 15 United States. In this study, we focus on a subset of these intake records in which the shelters indicated the intake was related to housing, *n* = 28,424.

These data were collected before, during, and after the height of the COVID-19 pandemic. Forty-three states, impacting all but one of the HASS Pilot shelters, introduced stay-at-home orders between March 1 and May 31, 2020. The extent of these orders in both strictness and how long they were effective varied greatly from state to state (33). In response to the possibility of a wave of evictions as renters lost jobs during the lockdowns, the Centers for Disease Control federal eviction moratorium for nonpayment of rent, effective between September 2020 and October 2021 (34). State and local eviction moratoria were also instituted, also varying greatly in content and timeline. Forty-four states implemented additional eviction moratoria effective between March 15, 2020 and at least June 30, 2021 (35). During this time period, shelter operations were also significantly impacted as many were forced to change their open-door policies to the community to appointment-based services (36). There is conflicting research on the impact of these changes on animal shelter intakes and outcomes, but, in general, current research suggests that relinquishments decreased and adoptions increased in the United States (37). Recently, these trends have reversed and news articles describe shelters across the United States as being “in crisis” as intakes have increased and adoptions have slowed, citing consequences such as reduced access to spay and neuter services during the COVID-19 pandemic, housing issues, and the “cumulative burden of higher costs of everything from groceries to rent” (38).

### 2.2 Measures

#### 2.2.1 Species

Types of animals were grouped into “dogs,” “cats” and “other,” which included any type of animal processed through the shelter that was not a dog or cat: rabbits, snakes, lizards, birds, chinchillas, ferrets, fish, guinea pigs, goats, hamsters, hedgehogs, rats, mice, opossums, pigs, raccoons, turtles, tortoises, and one wolf.

#### 2.2.2 Breed

Shelters reported the breed of dog, cat, and (when applicable) other animals. Two hundred and eight different dog breeds and breed mixes were contained in the dataset. We created an indicator variable for pit bull-type dogs, which are most commonly affected by breed-specific legislation (BSL) and are often specifically named as banned in apartment rentals. Pit bull-type dogs included in this indicator group were American Bulldogs, American Bullies, American Pit Bull Terriers, American Staffordshire Terriers, Dogo Argentinos, Presa Canarios, Staffordshire Bull Terriers, and any dog coded as a mix of the aforementioned breeds.

#### 2.2.3 Intake size

Animal sizes were categorized into small, medium, large, and extra-large upon intake by shelter staff. For the purposes of the analyses, we did not recode this variable. Animals categorized as small cats had a mean weight of 7.8 lbs., small dogs had a mean weight of 12.2 lbs., medium cats had a mean weight of 12.1 lbs., medium dogs 45.4 lbs., large cats 13.6 lbs., large dogs 74.5 lbs., and extra large cats 15.9 lbs., and extra-large dogs 92.0 lbs.

#### 2.2.4 Weight

The animal’s weight at intake was reported in pounds.

#### 2.2.5 Intake reason

The original intake reason consisted of 52 housing subtype intake categories. We condensed the categories into groups that were conceptually related: “loss of home” represented any intake reason related to evictions, foreclosures, and other housing loss; “unhoused” represented categories designating an unhoused owner; “restrictions” referred to any intake related to pet restrictions on housing, such as breed or size bans; “landlord” represented anything related to a landlord conflict; and “unspecified” was the catch-all category for anything related to housing or moving without additional descriptive details. Only a single reason for surrender was collected in these data.

#### 2.2.6 Age

The animal’s age was entered by shelter staff at intake.

#### 2.2.7 Outcome type

Categories for animal outcomes (i.e., how the animal left the shelter) were “admin, lost, missing or stolen,” “adopted,” “died in care,” “disposal,” “euthanasia,” “foster placement,” “return to owner,” “service out,” “trap neuter return (TNR),” transferred out,” and “wildlife out.” We created an indicator to represent animals that left the shelter alive (e.g., adoption, foster, transfer, etc.) versus those who died during their shelter stay (e.g., euthanasia, etc.).

#### 2.2.8 Length of stay

The amount of time, in days, that the animal was in the care of the shelter.

#### 2.2.9 Total intakes

The number of animal intakes per day, per shelter.

### 2.3 Analytic procedures

First, we obtained descriptive information for overall housing intakes, housing intake subtypes, housing-relinquished animal information, and housing-relinquished animal outcome information. Tests for multi-modality of weight variables were performed in two ways, first, using a more traditional DIP test for unimodality (39) was performed on the overall weight distribution for dogs and cats. For comparison purposes, we also assessed the weight distribution for the entire intake dataset. Follow-up analyses for weight on dogs and subsets of dog properties (outcome and breed, specifically pit bull-type breeds) were performed using Kernel Density Estimation via the scikit-learn package and local maxima peak detection using the Python SciPy package (40, 41). Bandwidth parameters were estimated using the Scott’s bandwidth (42) computed in statsmodels (43). We then used bivariate tests of association (chi-squared tests, Wilcoxon rank-sum/Mann–Whitney tests) to assess differences in age and length of stay by species (cat and dog only), as well as differences in outcomes by the animals’ characteristics. Next, we used multivariate logistic regression models to assess associations between animal characteristics and the odds of a live outcome (versus dead). In time series analysis, although there are ways to use ordinary least squares and linear regression to analyze the data, these often come with assumptions which cannot be satisfied by the data such as non-normality of the residuals. In order to address the question of whether increasing or decreasing trends are present in the time series data in this analysis, we instead leverage the Mann-Kendall (MK) test for monotonic trends (44, 45). This test makes one critical assumption that the signal is not autocorrelative. This assumption can be tested directly via a Durbin–Watson (DW) where values DW > 1 and DW < 3 indicate the assumption of the MK test is satisfied (46). All tests ran in this analysis satisfied this assumption, thus the MK test results can be interpreted directly. The MK test (in the two-tailed form used here) provides a measure of if the signal is increasing, decreasing, or has no trend. *p*-values and slopes are reported on column-wise normalized data to provide across-signal comparisons. In the multivariate models, observations with missingness on key variables were excluded from the analysis. Logistic regression assumptions were tested using Box-Tidwell regression (47). Any significant continuous variables that did not meet the linearity assumption were converted to categorical variables. All other regression assumptions were met. All analyses were conducted using Stata version 17 and Python 3.10.

## 3 Results

### 3.1 Descriptive findings

**Figure 1:**
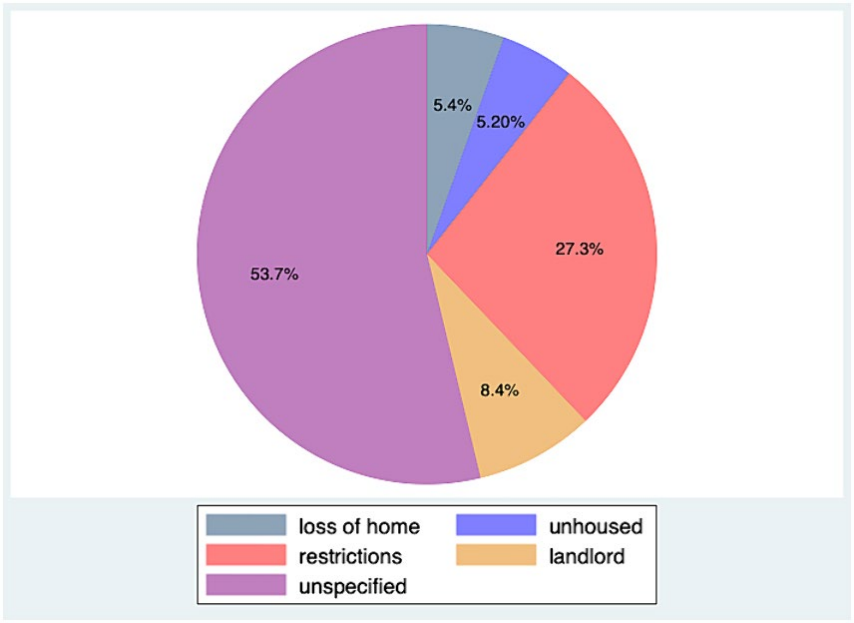
Housing intake subtypes as proportions of overall housing intake for all animals (dogs, cats, other; *n* = 26,715). Data from 21 municipal and non-profit animal shelters in 15 U.S. states.

Of the 1,021,204 total intake records, 14% (*n* = 28,424) were coded by the shelters as related to housing. Unspecified housing issues were the most common subtype of housing-related intake, followed by pet-related restrictions (e.g., breed and size bans), landlord issues, loss of housing, and unhoused owners (see Figure 1).

### 3.2 Types of animals relinquished due to housing issues

#### 3.2.1 Size and type

**Figure 2:**
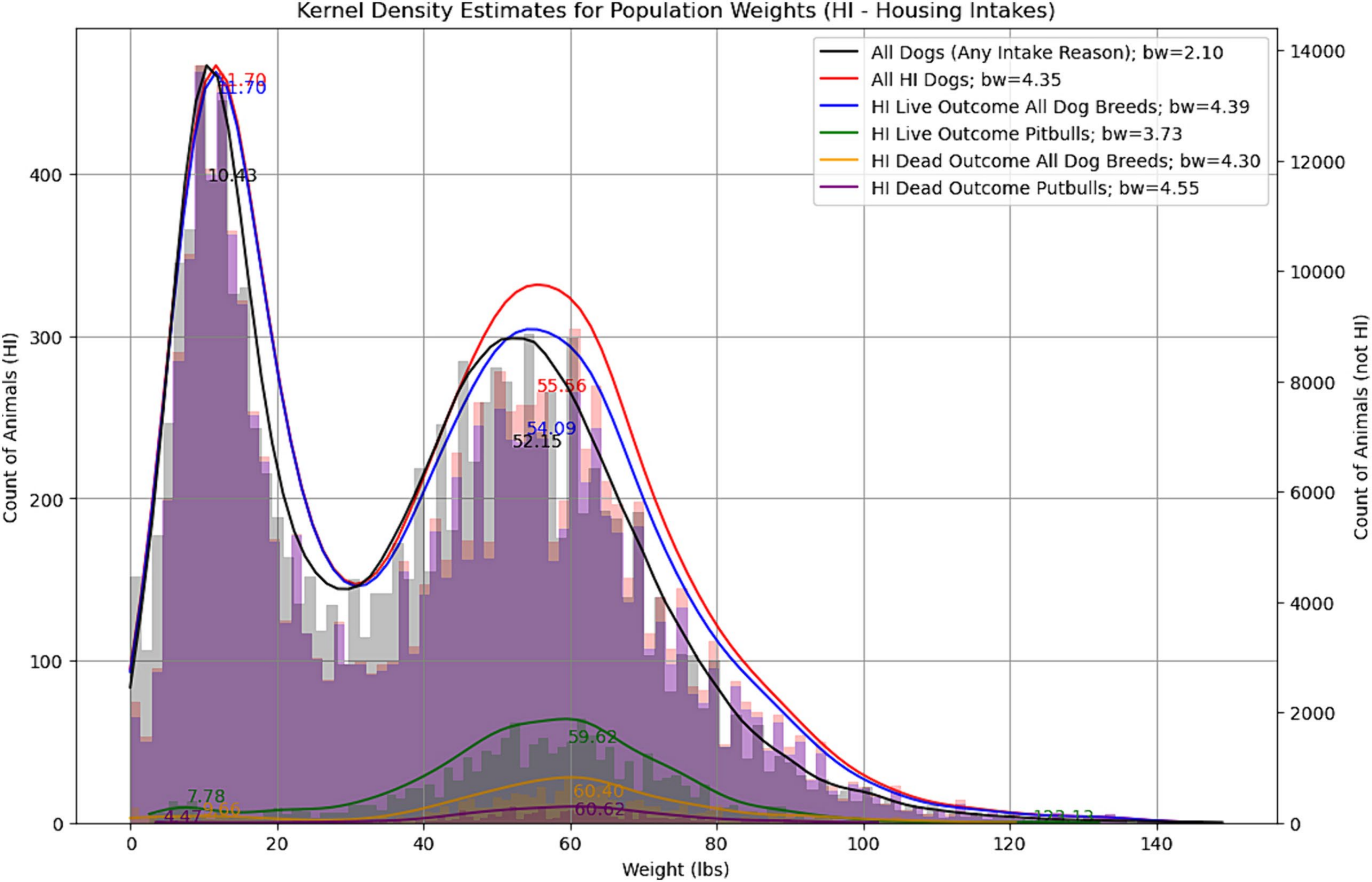
Dog weight at intake: all dogs regardless of intake reason (*n* = 344,209, black line); all housing-related dog intakes (*n* = 11,400, red line), by live (*n* = 10,821, blue line) or dead outcome (*n =* 572, yellow line), and by pit bull-type breed live outcome (*n* = 1,468, green line) and pit bull-type breed dead outcome (*n* = 198, magenta line) with corresponding density plots. Data from 21 municipal and non-profit animal shelters in 15 U.S. states.

Large dogs comprised the highest proportion of intakes (20%); however, small dogs were 19% of the intakes, and the weight distribution of the dogs was bimodal (DIP Test; *p* < 0.05) with peaks at 11 and 55 lbs. (see Figure 2). Medium dogs were 17%, followed by small cats (16%), medium cats (12%), large cats (7%), small other animals (3%), extra-large dogs (2%), medium other animals (2%), large other animals (1%), extra-large cats (1%), and extra-large other animals (<1%; there were only three total other type animals coded as extra-large: an iguana, a pig, and a wolf).

#### 3.2.2 Age

Intake age in days ranged from 0 to 8,402. The median age was 730 days; 25% of the animals were under a year old. Age varied significantly between cats and dogs: the mean cat intake (*n* = 10,455) age was 1,085 days and the mean dog intake (*n* = 15,715) age was 1,271 days (z = −20.498, *p* < 0.001). The median cat intake age was 609 days, and median dog intake age was 749 days.

#### 3.2.3 Dog breed

The most common breed relinquished was “mixed breed” (35%), followed by American Pit Bull Terriers (12%), Chihuahuas (5%), Labrador Retrievers (5%), and German Shepherds (4%). Pit bull-type dogs made up 16% of all dogs relinquished (*n* = 2,539). Twelve percent of the restriction-related intakes were pit bull-type dogs, whereas 27% of landlord-related relinquishments were pit bull-type dogs.

#### 3.2.4 Length of stay

The shortest length of stay was zero days and the longest was 766 days. The median length of stay was 6 days (this did not vary between cats and dogs); 25% stayed for 2 days or less. Length of stay varied significantly between cats and dogs: the mean length of stay for cats (*n* = 10,528) was 17 days, whereas the mean length of stay for dogs (*n* = 15,793) was 20 days (*z* = −4.147, *p* < 0.001).

### 3.3 Outcomes of housing-relinquished animals

#### 3.3.1 Live outcomes

Ninety-five percent of housing-relinquished animals had a live outcome: 76% adopted, 12% transferred out, 3% returned to the owner, 3% foster placement, 1% service and wildlife out, and a single record of TNR. These outcomes varied significantly by cats (*n* = 10,528) and dogs (*n* = 15,739; *X*^2^(10) = 578.37, *p* < 0.001): 82% of cats were adopted, 10% transferred out, 2% returned to owner, 2% foster placement, <1% service and wildlife out, and one TNR record. In terms of dogs, 72% were adopted, 13% transferred out, 4% returned to owner, 4% foster placement, <1% service and wildlife out, and none of the dogs had a TNR outcome.

#### 3.3.2 Non-live outcomes

The 5% non-live outcomes comprised primarily euthanasias (4.5%), and less than 1% of each lost, missing, or stolen, died in care, and disposal. Euthanasias were more common for dogs than cats: 6% of dogs and 3% of cats were euthanized. Pit bull-type dogs were more likely (13%) than other breeds (5%) to have a non-live outcome [*X*^2^(1) = 243.63, *p* < 0.001]. Pit bull-type dogs were also less likely to be adopted (67%) than other breeds (73%) and more likely to be euthanized (13%) than other breeds [5%; *X*^2^(10) = 349.38, *p* < 0.001]. However, pit bull-type dogs were more likely to be returned to their owners (5%) than other breeds (4%) and placed in foster care (6%) than other breeds [3%; *X*^2^(10) = 349.38, *p* < 0.001].

#### 3.3.3 Factors predicting live versus non-live outcomes

**Figure 3:**
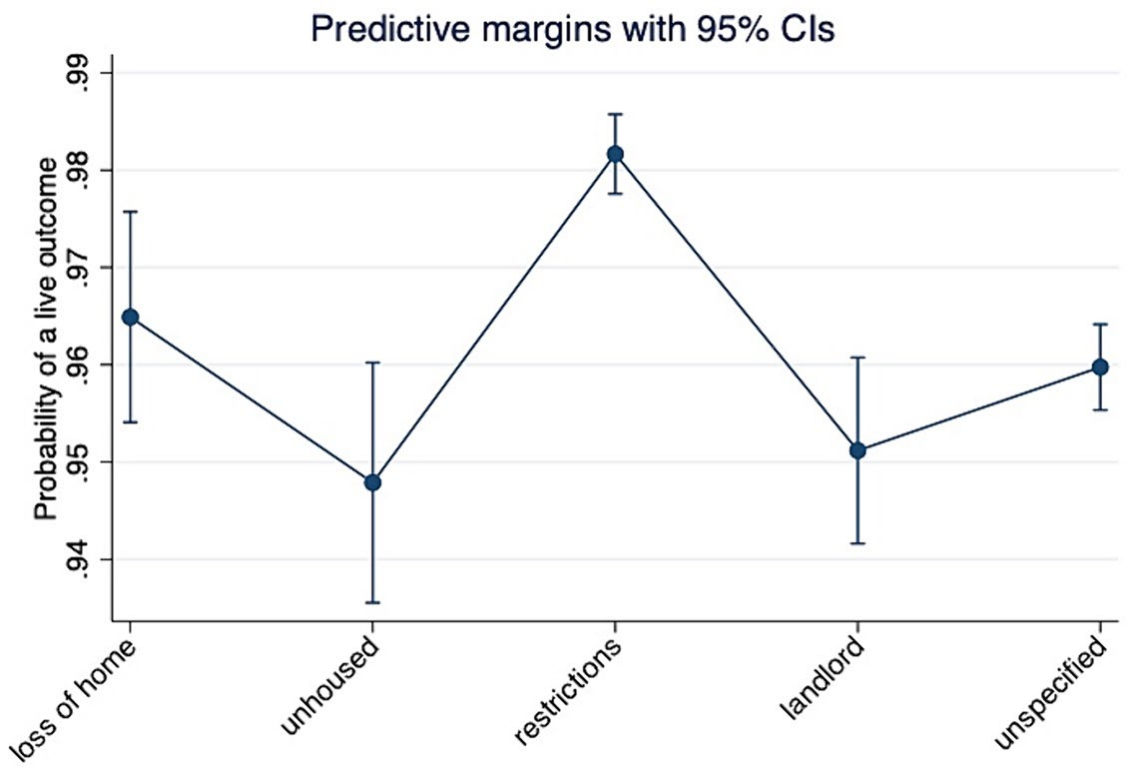
Probability of live outcome by housing intake subtype, adjusted for length of stay, age, size, and species (dogs, cats, other; *n* = 15,807). Data from 21 municipal and non-profit animal shelters in 15 U.S. states.

Unhoused owner intakes had the lowest live outcome odds (*n* = 15,807, *OR* = 0.64, *p* < 0.05) and restrictions intakes had the highest (OR = 1.99, *p* < 0.01), accounting for length of stay, age, species, and size (See Figure 3). Compared to small cats, small dogs had the highest odds of a live outcome (OR = 3.16, *p* < 0.001), while large dogs (*OR* = 0.58, *p* < 0.001) had the lowest. Compared to the youngest age group (0–8 months), those 1.44 years and above had lower odds of a live outcome (Q3: *OR* = 0.53, *p* < 0.01; Q4: *OR* = 0.53, *p* < 0.001; Q5: *OR* = 0.34, *p* < 0.001, see Table 1).

**Table 1:** Summary of logistic regression analysis for variables predicting likelihood of a live outcome.

Predictor	Odds Ratio	95% CI
Intake Subtype (ref = loss of home)		
Unhoused owner	0.64*	0.43–0.98
Restrictions	1.99**	1.33–2.98
Landlord issues	0.67*	0.45–0.98
Unspecified	0.89	0.63–1.26
Length of stay (in days)	1.00	0.99–1.00
Age quintiles (ref = Q1, 0–8 months)		
Q2, 8 months-1.44 years	1.03	0.71–1.51
Q3, 1.44–2.89 years	0.59**	0.41–0.83
Q4, 2.90–5.22 years	0.53***	0.38–0.75
Q5, 5.23–23.02 years	0.34***	0.25–0.48
Size/Species (ref = small cat)		
Medium cat	1.59*	1.08–2.33
Large cat	1.99**	1.23–3.24
X-Large cat	6.15	0.84–44.83
Small dog	3.16***	2.09–4.77
Medium dog	0.69*	0.52–0.94
Large dog	0.58***	0.43–0.77
X-Large dog	0.61	0.36–1.03
Small other	0.81	0.46–1.44
Medium other	3.72	0.90–15.33
Large other	1.53	0.47–4.95
Constant	48.25***	31.16–74.72
LR *X*^2^ (df)	344.66 (19)***	
Pseudo *R*^2^	0.07	
Pearson goodness-of-fit X^2^(df)	7464.71 (5722)***	
*N*	15,807	

Values shown in cells are odds ratios (OR). ***p < 0.001; **p < 0.01; *p < 0.05. Data from 21 municipal and non-profit animal shelters in 15 United States.

Among dogs, pit bull-type dogs had lower odds of a live outcome than other breeds, accounting for intake subtype, length of stay, and age (*n* = 14,636, *OR* = 0.37, *p* < 0.001; see Table 2).

**Table 2:** Summary of logistic regression analysis for variables predicting likelihood of a live outcome, dogs only.

Predictor	Odds ratio	95% CI
Intake Subtype (ref = loss of home)		
Unhoused owner	0.87	0.58–1.31
Restrictions	1.74**	1.22–2.47
Landlord issues	0.84	0.58–1.19
Unspecified	0.78	0.57–1.07
Length of stay (in days)	1.00	0.99–1.00
Age quintiles (ref = Q1, 0–8 months)		
Q2, 8 months-1.44 years	0.32***	0.22–0.48
Q3, 1.44–2.89 years	0.21***	0.14–0.31
Q4, 2.90–5.22 years	0.18***	0.13–0.27
Q5, 5.23–23.02 years	0.18***	0.13–0.27
Pit bull-type dog	0.37***	0.31–0.43
Constant	88.75***	55.89–140.92
LR *X*^2^ (df)	403.01 (10)***	
Pseudo *R*^2^	0.06	
Pearson goodness-of-fit X^2^(df)	3111.74 (2684)***	
*N*	14,636	

Values shown in cells are odds ratios (OR). ***p < 0.001; **p < 0.01; *p < 0.05. Data from 21 municipal and non-profit animal shelters in 15 United States.

### 3.4 Longitudinal trends in housing intakes

#### 3.4.1 Housing intake subtypes

Over time, intakes due to loss of home increased (*p* < 0.001, *z* = 9.82, *slope* = 0.29), while intakes due to pet restrictions (*p* < 0.001, *z* = −6.82, *slope* = −0.17) and landlord issues decreased (*p* < 0.001, *z* = −4.89, *slope* = −0.08). Longitudinal changes in unhoused intakes, unspecified housing intakes, and overall total housing intakes were not statistically significant.

#### 3.4.2 Live outomes

Over time, live outcomes of all housing-relinquished animals decreased (*p* < 0.001, *z* = −6.91, *slope* = −0.11).

#### 3.4.3 Geography

**Figure 4:**
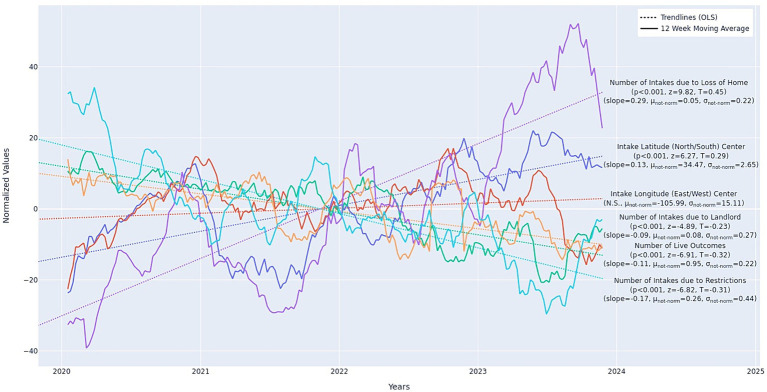
Longitudinal trends in housing-related intakes (dogs, cats, other) by: geographic latitude/longitude (*n* = 26,634), live outcome (*n* = 28,413), loss of home subtype (*n* = 1,451), landlord subtype (*n* = 2,240), restrictions subtype (*n* = 7,288). Values displayed in the figure are normalized. The non-normalized slopes and mean min/max values are as follows: latitude: slope = 0.003, min = 33.54, max = 35.95; longitude: slope = −105.99, min = −112.87, max = −99.68; live outcome: slope: 0.038, min = 21.0, max = 239.0; loss of home subtype: slope = 0.071, min = 21.0, max = 239.0; landlord subtype: slope = −0.021, min = 0.0, max = 63.0; restrictions subtype: slope = −0.07, min = 3.0, max = 101.0. Slopes are in units Number of Intakes due to <Reason> per Week and Longitude/Latitude Degrees per Week. Slopes are best-compared relative to one another when units are shared. Longitude is not significant but is provided for completeness as Latitude is significant. Minimum and Maximum weekly values are also provided for reference. Data from 21 municipal and non-profit animal shelters in 15 United States. Twelve-week moving average omits the first 11 weeks of the data.

In terms of geographic region, there was no statistically significant change in intake rates by east/west comparisons. However, housing intake frequencies shifted north from south over the study period (*p* < 0.001, *z* = 6.27, *slope* = 0.13). See Figure 4.

#### 3.4.4 Animal characteristics

**Figure 5:**
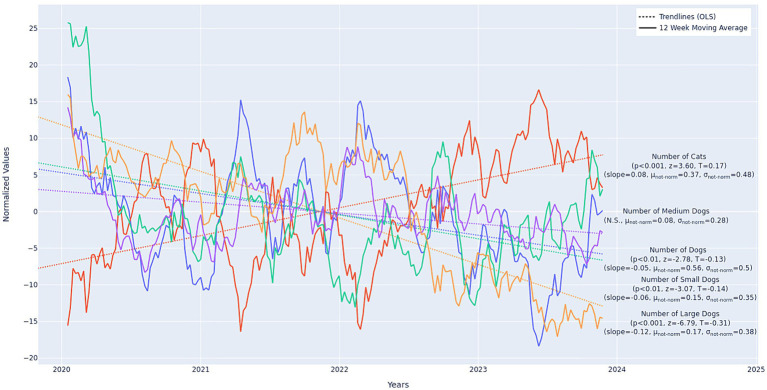
Longitudinal trends in cat (total cats *n* = 10,530) and dog (medium dogs *n* = 2,415, total dogs *n* = 15,802, small dogs *n* = 4,151, large dogs *n* = 4,845) housing intakes. Values displayed in the figure are normalized. The non-normalized slopes and mean min/max values are as follows: cats: slope = 0.06, min = 3.0, max = 100.0; all dogs: slope = 0.004, min = 13.0, max = 136.0; medium dogs: slope = 0.085, min = 0.0, max = 29.0; small dogs: slope = −0.01, min = 1.0, max = 49.0; large dogs: slope = −0.04, min = 2.0, max = 52.0. Slopes are in units Number of <Animal Category> per Week increase (for positive values) or decrease (for negative values). Slopes are best-compared relative to one another when units are shared. All slopes displayed in this figure should be considered “small” (though significant) in magnitude. Minimum and Maximum weekly values are also provided for reference. Note that Medium dogs are included for completeness, though they were a non-significant category. Data from 21 municipal and non-profit animal shelters in 15 United States. Twelve-week moving average omits the first 11 weeks of the data.

The frequencies in sizes and species of housing-relinquished animals also changed over time. Overall cat intakes increased (*p* < 0.001, *z* = 3.60, *slope =* 7.34), while dogs decreased over the study period (*p* < 0.01, *z* = −2.78, *slope* = −0.05). Among dogs, large dog intakes decreased at the fastest rate (*p* < 0.001, *z* = −6.79, *slope* = −0.12), small dogs at a slower rate (*p* < 0.01, *z* = −3.07, *slope* = −0.06), and medium dogs decreased slightly, but not significantly. See Figure 5.

**Figure 6:**
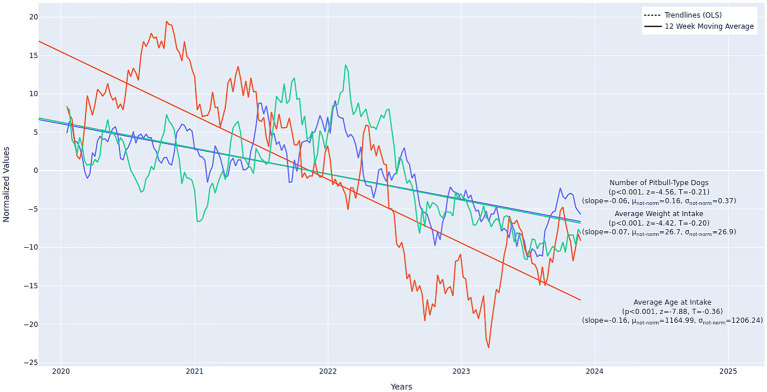
Longitudinal trends in housing intakes by pit bull-type dogs (*n* = 2,539), average weight (*n =* 20,507), and average age (*n* = 28,036). Values displayed in the figure are normalized. The non-normalized slopes and mean min/max values are as follows: pit bull-type dogs: slope = −0.02, min = 0.0, max = 32.0; average weight: slope = −0.02, min = 15.62, max = 39.02; average age: slope = −1.65, min = 762.51 days, max = 1,602.34 days. Slopes are in units Number of Pitbulls per Week, Average Weight in Lbs per Week, and Average in Age in Days per Week. Slopes are best-compared relative to one another when units are shared, therefore the values in this figure should be compared to the other longitudinal figures, not among themselves. The number of pit bull-type dogs per week and average weight, though significant, should be considered “small” in magnitude, while age, trending toward younger animals, should be considered “moderate” to “large” in magnitude. Data from 21 municipal and non-profit animal shelters in 15 United States. Twelve-week moving average omits the first 11 weeks of the data.

The number of intakes that were pit bull-type dogs (compared to all other breeds) decreased over time (*p* < 0.001, *z* = −4.56, *slope* = −0.06), as did average animal weight (*p* < 0.001, *z* = −4.42, *slope* = −0.07) and age (*p* < 0.001, *z* = −7.88, *slope* = −0.16). See Figure 6.

## 4 Discussion

In this study, we analyzed a large dataset of shelter intakes from 21 animal shelters across the U.S. from 2019–2023. We first described the share of shelter intakes related to housing issues, the characteristics of the animals relinquished due to housing, the outcomes of the housing-relinquished animals, and longitudinal trends in housing-related intakes. We discuss our findings and their implications in the following paragraphs.

Overall, housing-related intakes made up 14% of all intakes in the dataset. This is similar to recent estimates in the Canadian province of British Columbia (20) and the United States (48), and lower than older estimates from the United States [e.g., (49)]. However, more recent data suggests that housing-related intake rates may vary from 7 to 33% of overall intakes across several shelters in the United States (50), implying that there may be regional or local impacts to consider. More research is needed to better understand how state and local housing policies and localized housing markets impact pet relinquishment. The unspecified housing subtype was the most common in this dataset, indicating an additional area for future research to better understand vague housing relinquishment reasons, the potential multifaceted nature of circumstances that lead to relinquishment, and to support ongoing efforts by shelters to collect more detailed, consistent intake information when pets are relinquished.

Large and small dogs made the highest proportions of housing-related intakes in terms of size and species. This roughly followed the distribution of overall intake weight regardless of reason during the same time period but with relatively higher intakes for at-risk populations such as pit bull-type dogs. Cats made up more than one-third, or 37%, of animals relinquished due to housing issues. Cats may be easier to keep in housing without landlord permission; however, undeclared pets bring an added risk of involuntary moving (3). In terms of dogs, mixed breed and pit bull-type dogs were the most common in this dataset. In Graham et al.’s 2018 study, owners of pit bull-type dogs faced added difficulties in searching for and securing rental housing (2). However, that one-third of all animals relinquished due to housing are cats, and that small dogs (with a peak at 11 pounds) are the second most impacted group of dogs, indicates a need to look beyond typically discussed breed, weight, and size barriers and explore more inclusive solutions to mitigate impacts on smaller dogs and cats. The perception that large dogs are the hardest to house prevailed to such an extent in Graham et al.’s (2) study that tenants opted for smaller-sized dogs because they were renting and anticipated facing restrictions on the size of pets as a result. These perceptions and preferences for smaller-sized dogs in rental housing are evident in lists of ‘top apartment dogs,’ even though many large dogs are suitable for apartment living.

The shelters represented in this dataset had an overall short length of stay and very high live outcome rate: 95% on average for housing-related intake animals. Pet-restriction-related intakes and animals who were young and/or small had the highest probability of a live outcome; however, unhoused owner intakes, pit bull-type dogs, and all animals above puppy to young adult age were the least likely to have a live outcome. While the health of pets of unhoused owners do not appear any different than that of housed pets (51), their lower live outcome rate suggests there is a gap in research on this topic. Dogs living with unhoused people tend to spend a significant portion (if not all) of their time outside and with their owner. Future research should assess underlying causes of a lower live outcome rate for these pets, including whether dogs relinquished by unhoused owners experience particular difficulty transitioning to a shelter environment where they spend a majority of their time indoors, without direct human contact, and in kennels. Regardless, these results certainly support the need for increased programming specifically targeted at keeping unhoused people and their pets together, both for the sake of this important human-animal bond, but also to prevent euthanasia of this particular group of pets. A vast majority (88%) of all animals were either adopted or transferred out to other rescue organizations, indicating a strong reliance on traditional animal sheltering strategies for preventing euthanasia of healthy, adoptable pets, but also indicating a potential gap in supportive services for keeping pets and people together when housing is the root of a person’s decision to relinquish their pet.

Longitudinally, we found evidence for an increase in intakes due to the loss of one’s home and decreases in intakes due to both pet restrictions and landlord issues. This could imply that the culture and policies around pet-inclusive housing are improving while overall housing insecurity is worsening. Indeed, housing costs, evictions, and homelessness have all been on an upward trend in recent years (30). Over the study period, we also found a shift in housing-related intakes from southern latitudes toward northern latitudes, but no similar trend east/west. This could reflect some combination of increasing housing affordability and cost of living issues in northern parts of the United States Data from The Council for Community and Economic Research indicates that, in general, more northern and coastal regions have higher costs of living — which includes housing costs. United States migration trends in recent years have shown an exodus from more northern, high-cost-of-living regions to primarily southern states, like Texas and Florida (52). Affordability and cost-of-living trends do not follow unidirectional East-to-West–or *vice-versa*–trends; instead, these issues are most notable on the coasts (53) and, generally, are less of an issue in the middle of the United States, which may explain the lack of significant trends over time longitudinally. In this data set, unspecified “moving” intake reasons were not separately analyzed. More research is needed to understand how United States migration patterns and regional changes in the cost of living and housing affordability are impacting pet-owning families and animal shelters.

In this dataset, dog intakes decreased while cat intakes increased, and among dogs, both large and small dogs, and pit bull-type breeds, each decreased significantly. This, like the intake subtypes, could be a result of dog-inclusive policies and culture, including repeals of breed-specific legislation in some metropolitan areas during the study period, i.e., Denver in 2021 and Miami in 2023. Additionally, average weight and age both decreased for all animals over the study period, potentially as an artifact of some of the COVID-19-related closures of spay/neuter programs (54), as well as in relation to the overall shift in relinquishment trends toward cats and away from large dogs. Notably, overall live outcomes of the housing-relinquished animals decreased over the study period, possibly reflecting broader trends in shelter overpopulation.

### 4.1 Implications for policy and practice

The data supports existing policy efforts to remove breed, weight, and size restrictions in rental housing policies. That pit-bull type dogs are relinquished and experiencing non-live outcomes at higher rates and continue to be adopted at lower rates relative to other dogs, further supports ongoing policy efforts to remove BSL in both the public policy arena and in private housing policies. The recent successful effort to add “XL Bully Breeds” to the Dangerous Dogs Act of 1991 in the United Kingdom is concerning for the significant progress made in the United States addressing the misplaced fear toward pit bull-type dogs (55).

These results also suggest that broader advocacy related to affordability and arbitrary restrictions on the number of animals allowed in units are likely necessary to address companion animal relinquishment on a broader level. Cats and small dogs, which are largely unaffected by typical breed, weight, and size restrictions, make up a significant portion of relinquished animals in this study and most current policy efforts are not sufficient to protect these groups of pets. Notably, while dog relinquishments have decreased over time, cat relinquishments have increased. Cat owners, in particular, may be subject to unjustifiable pet rents. The rental housing industry has argued that while pet security deposits and nonrefundable fees are utilized in the case of damage or upkeep to a rental unit (similar to a traditional security deposit), pet rents are used to cover the costs of public amenities like dog parks, pet washing areas, and pet waste stations [e.g., (56, 57)], none of which are justifiable to charge cat owners who likely do not utilize any of these common-area amenities.

These data also suggest that arbitrary restrictions on the number of animals allowed in rental units likewise warrant further attention from policy advocates and policymakers. This has been a long-standing issue in discussions of municipal ordinances imposing arbitrary restrictions on the number of pets allowed in a household (58). At the ordinance level, proponents of limiting the number of pets a person can have in their unit suggest that these limits are needed to prevent hoarding situations and to protect against noise or odor issues that may be associated with the ownership of multiple animals. However, opponents of arbitrary pet limits argue that negligence and/or nuisance laws exist to ensure that conditions related to pet ownership do not become detrimental to others’ use and enjoyment of their own property and that different households can care for different numbers of animals [e.g., (59)]. Although more research is needed to understand the impact of pet number limits in rental housing on different species and sizes of dogs, there is some evidence to suggest that limits to two pets may disparately impact cat owners. Sources range from an average of 1.76 (60) a to an average of 2.1 cats (61) per household, whereas data about households with dogs state an average of 1.46 dogs per household (60), thus more cat-owning households than dog-owning households may be impacted by these limits. Pet-inclusive housing advocates may benefit from utilizing similar arguments as those relied on in the ordinance space: arbitrary number limits are unlikely, alone, to mitigate concerns about damage, noise, odor, etc., and local negligence and nuisance laws may be the appropriate avenues for addressing these issues related to pet ownership.

In this study, only 4% of dogs were returned to their owners, while a vast majority (88%) were either adopted out or transferred to rescue organizations. Although it’s unclear from the data what percentage of owners would have wanted to keep their pet, research suggests that tenants who have to choose between their pet or a place to live face devastating choices and that housing-related relinquishment is the most-cited reason for involuntary relinquishment (62). Further, 2023 research from the Pew Research Center suggests that 97% of pet owners consider their pets family (63). Taken together, this may indicate that more people want to reunite with their pets than were able to in this study. Shelters represented in this study are utilizing their traditional animal sheltering tools very well (adoption to new families and well-established transfer partnerships), but both shelters and funding sources (government agencies, foundations, etc.) likely have room for incorporating some of the more progressive programming meant to keep pets with their original families, including temporary fostering, landlord-intervention programs, connecting pet owners to legal services for housing support, and programs providing for resources like pet fee and deposit assistance.

### 4.2 Limitations and future directions

Several limitations to this study should be noted. First, the data were collected across 21 shelters over a five-year period, which inherently comes with some potential data consistency issues. For example, one shelter may not consider dog barking to be related to housing and code the intake reason as behavioral, while another shelter may recognize it as moving. This issue is likely reflected in our fit statistics for the multivariate regression models: overall fit was somewhat poor and thus the results should be interpreted with some caution. However, this issue is extremely common and expected for these types of data and impacts interpretation of the results in that we know these effects are smaller and more diffuse across this highly heterogenous dataset. Additionally, only one relinquishment reason was recorded per intake record, which did not allow us to assess the known complexity of animal relinquishment. Relatedly, the “unspecified” category subtype was more than half of all intakes; these intakes were coded simply as “housing issues” or something similar and thus we could not garner more specific information from those records. Furthermore, due to variability between staff and shelters and the administrative nature of these data, there may be differential misclassification bias. And, importantly, due to our data use agreement with HASS, we were only able to report data in aggregate, which could result in missing some possible shelter-level variance in intakes, outcomes, and animal characteristics.

Second, visual identification of dog breeds is known to be extremely unreliable, even by animal professionals and shelter staff (64, 65). Further, shelters may avoid using specific breed labels due to breed-specific legislation (i.e., “dangerous dog” laws) and public perceptions, particularly when the dog is suspected to be a pit bull-type breed. Third, our dataset is not without bias as the records provided are from shelters with the resources to devote staff time to working with HASS and providing consistent data. These shelters represent a higher live release rate than national averages, which is estimated to be closer to 88% as of 2023 (66) potentially due to their participation in progressive shelter management policies. However, to our knowledge, the dataset we analyzed in this study is the best current documentation of detailed housing-related relinquishment in such high numbers. Future studies should work with sheltering organizations to bolster data collection and consistency efforts. Fourth, the time period of the dataset represents before, during, and after the height of the COVID-19 pandemic. There were many housing policy and sheltering operational changes over this timeframe that would have impacted longitudinal trends in housing-related intakes. Finally, we did not explore interactions among independent variables in the regression models in this study; because most predictors were significant, future research should consider moderation models via interaction or stratified regression to further examine group-level effects and better assess relationships among the variables that were not explained by the models in the current manuscript.

## 5 Conclusion

In this study, we analyzed over 28,000 intake records related to housing from 21 animal shelters across the United States between 2019–2023. We found that 14% of all relinquishments in the database were housing-related, which included the subtypes of unspecified housing, pet-related restrictions, landlord issues, housing loss, and unhoused owners. Among these, the most frequently surrendered animals due to housing constraints were large and small dogs, pit bull-type dogs, and mixed-breed dogs. Live outcomes (e.g., adoption) were very common in this dataset overall, but pit bull-type dogs and animals relinquished by unhoused owners were more likely to die in the shelter (primarily via euthanasia), and live outcomes were on a decreasing trend among all housing-relinquished animals. Over the duration of the study, instances of animals entering shelters due to loss of housing rose, while those due to pet restrictions and landlord conflicts declined. Moreover, there was an increase in cat intakes and a decrease in dog intakes observed over the study period. The proportion of pit bull-type dogs among all breeds that were housing-relinquished declined over time, as did the average weight and age of animals upon intake. Taken together, our results suggest that the culture around pet-inclusive housing may be improving while broader housing insecurity is worsening. To better prevent companion animal relinquishment due to housing issues and inclusively support the human-animal bond, invested parties should promote progressive housing and social policies both in their state as well as federally.

## Data availability statement

The data analyzed in this study is subject to the following licenses/restrictions: Data are available upon reasonable request from LL. Requests to access these datasets should be directed to lauren.loney@austinpetsalive.org.

## Author contributions

JA: Conceptualization, Formal analysis, Investigation, Methodology, Project administration, Resources, Supervision, Visualization, Writing – original draft, Writing – review & editing. LL: Conceptualization, Data curation, Resources, Writing – original draft, Writing – review & editing. KH: Conceptualization, Data curation, Formal analysis, Investigation, Methodology, Visualization, Writing – original draft, Writing – review & editing. TG: Conceptualization, Investigation, Writing – original draft, Writing – review & editing.
